# Using democracy to award research funding: an observational study

**DOI:** 10.1186/s41073-017-0040-0

**Published:** 2017-09-15

**Authors:** Adrian G. Barnett, Philip Clarke, Cedryck Vaquette, Nicholas Graves

**Affiliations:** 1 Institute of Health and Biomedical Innovation, Queensland University of Technology, 60 Musk Avenue, Kelvin Grove, QLD 4059 Australia; 2 Melbourne School of Population and Global Health, University of Melbourne, Melbourne, VIC Australia

**Keywords:** Peer review, Research funding, Research fellowships, Meta-research

## Abstract

**Background:**

Winning funding for health and medical research usually involves a lengthy application process. With success rates under 20%, much of the time spent by 80% of applicants could have been better used on actual research. An alternative funding system that could save time is using democracy to award the most deserving researchers based on votes from the research community. We aimed to pilot how such a system could work and examine some potential biases.

**Methods:**

We used an online survey with a convenience sample of Australian researchers. Researchers were asked to name the 10 scientists currently working in Australia that they thought most deserved funding for future research. For comparison, we used recent winners from large national fellowship schemes that used traditional peer review.

**Results:**

Voting took a median of 5 min (inter-quartile range 3 to 10 min). Extrapolating to a national voting scheme, we estimate 599 working days of voting time (95% CI 490 to 728), compared with 827 working days for the current peer review system for fellowships. The gender ratio in the votes was a more equal 45:55 (female to male) compared with 34:66 in recent fellowship winners, although this could be explained by Simpson’s paradox. Voters were biased towards their own institution, with an additional 1.6 votes per ballot (inter-quartile range 0.8 to 2.2) above the expected number. Respondents raised many concerns about the idea of using democracy to fund research, including vote rigging, lobbying and it becoming a popularity contest.

**Conclusions:**

This is a preliminary study of using voting that does not investigate many of the concerns about how a voting system would work. We were able to show that voting would take less time than traditional peer review and would spread the workload over many more reviewers. Further studies of alternative funding systems are needed as well as a wide discussion with the research community about potential changes.

**Electronic supplementary material:**

The online version of this article (doi:10.1186/s41073-017-0040-0) contains supplementary material, which is available to authorized users.

## Background

Peer review has long been criticised for being inaccurate, costly, opaque, prone to abuse and a barrier to innovation [1–3]. Biases in peer review have been demonstrated in experiments and observational studies [4, 5]. Despite its failings, peer review is used almost ubiquitously for deciding what papers are published and what grant applications win funding.

The typical peer review process for funding applications requires large amounts of time and effort from scientists and administrators [6]. Many scientists put their research on hold for months to complete lengthy application forms [6]. It can be a frustrating process given that the outcomes are somewhat random [7] and low success rates mean that excellent researchers and proposals are often not funded. The high costs and uncertain returns of current funding systems has led to calls for alternative systems to be developed [8], and many of the issues with winning research funding have been recognised for more than 30 years [9]. Some funders of health and medical research have responded to concerns around the time pressures for applicants by changing their processes, including in the USA [10], Canada [11] and Australia [12].

Applicants’ time is important for funding systems because there are large opportunity costs to completing application forms, especially when success rates are low. Our previous research in Australia estimated that 510 years of research go into unsuccessful applications, which is the equivalent of funding 510 fellowships [13]. Some funding systems have been shown to be so inefficient that the cost of applying outweighs the amount of money awarded [14], and some funding systems may collect too much information which swamps reviewers and hinders decision-making [6]. The time spent on applications can spiral out of control when funding becomes hyper-competitive [15], and even shortening the length of the application may not reduce the time that researchers spend [13].

Some researchers have expressed strong cynicism about the current funding system, as shown by this recent quote from a peer review panel member: “It’s really virtually impossible to write an Australian Research Council grant now without lying” [16]. The randomness of the current system is also a commonly cited complaint amongst researchers, and a previous qualitative study included this admission from a panel member about the decision-making process: “There’s an awful lot of chance whether you go above the line or below the line” [17]. Our previous survey of over 200 health and medical researchers in Australia found that 74% agreed with the statement, “I submit proposals each year because chance is involved in being funded” [18]. We also found elements of subverting the system as 46% agreed with the statement, “I have already done more than 25% of the work proposed in my submitted research plan” [18]. However, this survey did also uncover benefits to the application process as 81% agreed with the statement, “If my proposal is unsuccessful, I still gain benefit from reading the literature and developing my scientific ideas in the proposal” [18].

### Alternative systems

Alternative systems have been suggested to replace traditional peer review, and we highlight five in Table 1. For a more detailed comparison of funding systems, see the review [19]. No funding system will ever be perfect, and hence, we are selecting the “least worst” system [20]. Compared with the traditional peer review, all the five systems in Table 1 are more transparent [21] and take much less time for applicants. None of these systems have yet to be tested in a large trial using real funding, although the New Zealand Health Research Council are using random funding [22].

**Table 1:** Some characteristics of five alternative models for funding research that do not require detailed applications

System	Benefits	Problems
Equal allocation	•Avoids peer review biases [8]	•Cannot fund higher cost research
Lottery	•Can increase efficiency by funding riskier research that would rarely be funded by traditional peer review [37]	•Politically problematic [38]
Automated scores	•Harnesses large amounts of existing data on researchers	•Can be gamed [39] •Takes no account of career disruption •Scores may have poor sensitivity and specificity
Prediction markets [33]	•Extracts more accurate information by paying reviewers proportional to their ability	•Reviewers may be lobbied to give good predictions •Potentially more suited to rating departments rather than individual researchers
Peer-to-peer distribution [23]	•Harnesses existing knowledge •Scientists with the greater respect of their peers have increased decision-making power	•Vulnerable to collusion •Results may be quite different in the first few years as the distribution mechanism stabilises

In this paper, we examine a democratic funding system, where a country’s research community vote every year in a secret ballot for those researchers that they think most deserve funding. The votes would be ranked, and the researchers funded in order until the funding pool was spent. Funding could be stratified by fields, national priorities and stage of career in order to meet national research goals. As per the current system in Australia, researchers would self-nominate their own fields and career stage, with verifications made by their own institution.

Democracy would be a potentially low cost system. Long applications would not be required, and the review workload would be shared by a nation’s entire scientific community. It would harness small amounts of local knowledge and amass this knowledge into a nationwide ranking of quality. It would still use peer review, but with many peers that have little individual influence. A democratic system is similar to the peer-to-peer distribution (Table 1), but voters have equal power over time rather than potentially gaining power over time by accumulating funding to redistribute [23].

There are many potential problems to a democratic system. It may become a popularity contest with results based on self-promotion ability rather than scientific ability. Some researchers may ignore ability and simply vote for their friends. Some researchers may rationally allocate many weeks or months lobbying for votes at the cost of their research. Researchers at larger institutions may be advantaged by getting more votes from colleagues and having access to better lobbying from large and well-organised administrative departments.

The aim of this study was to examine some of these issues using a mock democratic funding system that used a convenience sample of Australian researchers.

## Methods

We used an online survey to collect votes from Australian researchers (see Additional file 1). The survey was piloted in January 2015 and the responses included in the study as the survey did not change. In April 2015, the survey was e-mailed to research administration offices around the country with a request to distribute the survey to their researchers (see Additional file 2 for an example). We also e-mailed colleagues directly using our existing contacts. The study was advertised on Twitter and was featured in an article in *The Conversation* [24]. Respondents were also encouraged to pass the survey on to other researchers.

Respondents were asked to name the 10 researchers currently working in Australia that they thought most deserved funding for future research. They were asked to rank them from highest to lowest. Naming fewer than 10 was permitted and all votes were counted. They were also asked to add the researcher’s current institution. All names were checked by an author (AGB) and a research assistant, and there were 55 occasions (5.7%) where a name was edited because of a spelling error. Responses were also edited to use consistent institution names by turning institution acronyms into full names. Respondents were asked for their current position and institution and how long it took them to think of the names. There was an optional comment box.

For simplicity, we use the language of voting, and so we refer to votes and voters (researchers) and use “ballot” or “ballot paper” for the online list of 10 votes.

To focus on the current Australian research community—who should have the best knowledge concerning who to vote for—we excluded votes from non-researchers such as university administrative staff (22 voters), those not currently working in research (9 voters) and retired researchers (2 voters).

For comparison, we used data on fellowships awarded by the two largest Australian research funders that use a competitive peer review process: the Australian Research Council (ARC) and National Health and Medical Research Council (NHMRC) for the years 2011 to 2014. We did not include project funding as a voting system concerns people, and therefore, fellowships are the most comparable category. For the ARC, we used Australian Laureate Fellowships and Future Fellowships. For the NHMRC, we used Career Development Fellowships, Established Career Fellowships and Research Fellowships.

The first page of the online survey was a participant information sheet, and participants needed to tick a box to agree to participate. This consent process and the study were approved by the Queensland University of Technology Research Ethics Unit.

### Statistical methods

We did not impute missing data and used all available responses for each question. The patterns and numbers of item missing data are in Additional file 2, and there was little missing data apart from the data lost because of those who started the survey but did not vote.

### Time taken

We used the median and inter-quartile range as descriptive statistics for vote counts and self-reported voting times. We used linear regression to estimate the extra time needed per vote, using the total number of votes per ballot and the time taken to complete the ballot.

We estimated the time a national voting system would take by extrapolating our survey results to the number of eligible researchers using the equation:

$$ \mathrm{National}\ \mathrm{voting}\ \mathrm{t}\mathrm{ime} = \mathrm{Number}\ \mathrm{o}\mathrm{f}\ \mathrm{national}\ \mathrm{researchers} \times \mathrm{Voting}\ \mathrm{response}\ \mathrm{rate} \times \mathrm{Average}\ \mathrm{t}\mathrm{ime}\ \mathrm{t}\mathrm{o}\ \mathrm{vote} $$

The number of national researchers was estimated by adding together the total number of users of the ARC (85,299 users) and NHMRC (35,447 users) online submission systems. Combining these two major national funders should include every active researcher in Australia. To adjust for overlap, we reduced the total by 10%. This reduction was based on finding the overlap in names of 2142 recent winners of ARC funding and 18,857 recent winners of NHMRC funding. We reduced the total number of researchers by a further 10% to adjust for non-Australian researchers. This was an estimate based on our own experience, as there were no data available to easily identify overseas researchers.

We estimated the voting response rate using the percent of respondents who started our survey and cast one or more votes. The average time taken to vote was calculated from our survey. We used a bootstrap procedure to include the statistical uncertainty in the response rate and average time; hence, the final estimate is presented as a mean time and 95% confidence interval. We converted the total time to working days using a 7.5-h day.

To estimate the number of votes in a national system, we multiplied the estimated number of voters by the number of votes per ballot from our survey. We bootstrapped the number of votes using the empirical distribution from the survey.

We compared the voting time with the peer review time used by the current fellowship system using the equation:

$$ \mathrm{National}\ \mathrm{t}\mathrm{ime}\ \mathrm{t}\mathrm{raditional}\ \mathrm{peer}\ \mathrm{review} = \mathrm{Number}\ \mathrm{o}\mathrm{f}\ \mathrm{applications} \times \mathrm{Average}\ \mathrm{t}\mathrm{ime}\ \mathrm{t}\mathrm{o}\ \mathrm{peer}\ \mathrm{review} $$

For the number of applications, we used the most recently available numbers for NHMRC Career Development Fellowships (431), NHMRC Research Fellowships (246), ARC Future Fellowships (315) and ARC Laureate Fellowships (115), a total of 1107. We used our previous estimates of the time needed to peer review a grant application, although this was for project funding not fellowship funding [25].

### Vote winners

We compiled votes using two methods: unweighted, based on the raw number of counts, and weighted, where the highest ranked researcher won 10 votes, the second 9 and so on. If a ballot contained fewer than 10 votes, then the highest ranked researcher still won 10 votes. We tabulated votes and highlighted the top 10 researchers. We e-mailed these top 10 researchers and asked them for permission to use their name. Nobody refused, but some researchers did not reply and we just give their gender.

To examine if voters favoured their own institution, we split each ballot into the number of votes for the home institution and number for other institutions. We then calculated the expected number under the null hypothesis of no favouring by multiplying the number of votes in each ballot by the overall proportion of votes for that institution. We then used a chi-squared test based on the difference in observed and expected votes and also give summary statistics for the difference in observed minus expected votes.

We compared the gender split of votes with recent fellowship winners using a logistic regression model with the votes for women expressed as a rate ratio rather than an odds ratio.

To examine voting patterns, we used a network diagram to link votes from the same ballot [26]. For example, if a ballot had three votes for researchers McGrew, Cuthbert and Dibble, then the network diagram would include three connections McGrew–Cuthbert, McGrew–Dibble and Cuthbert–Dibble. The diagram was drawn using the Fruchterman–Reingold algorithm.

## Results

### Time

**Fig. 1:**
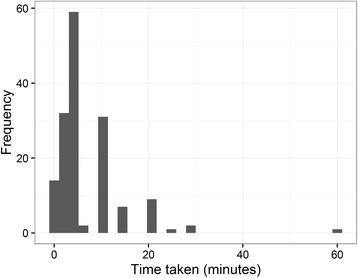
Histogram of time taken to cast votes (*n* = 153)

The distribution of voting times is in Fig. 1. The median time to cast the votes was 5 min (inter-quartile range 3 to 10 min). Each additional vote took an additional 32 s on average (95% CI 8 to 57 s).

A national system would involve an estimated 37,626 voters (95% confidence interval 33,052 to 42,121) and would take 599 working days (95% confidence interval 490 to 728). This many voters would cast 249,000 votes (95% confidence interval 215,000 to 284,000).

The current system of peer review for fellowships takes an estimated 827 working days to review 1107 applicants. This is based on a review time of 5.6 h per application.

### Vote winners

The total number of voters was 169 with votes cast between 27 Jan. 2015 and 27 Apr. 2015. We cannot calculate a response rate as we used a convenience sample. A large number of respondents started the survey but did not complete the votes. Of the respondents who started the survey, there were 271 (62%) who did not vote and 169 (38%) who did.

There were 1119 eligible votes. The median number of votes per ballot paper was 6 (inter-quartile range 4 to 10). There were no ballots where the same researcher was named twice. We could not count the number of researchers voting for themselves (as the survey was anonymous), but one researcher mentioned they did this in the comment box.

The median number of institutions per ballot paper was 4 (inter-quartile range 2 to 6). Researchers were more likely to vote for colleagues from their own institution. The median number of extra votes for their home institution was 1.6, with an inter-quartile range of 0.8 to 2.2 (chi-squared *p* value <0.001). Only two (3%) ballots contained only votes for the voter’s institution.

The top 10 researchers using vote numbers and weighted votes are in Table 2. The top 10 included two of the study authors, which could be somewhat due to “friendly” voting as the survey was distributed to researchers using our established networks. The top 10 also included well-known Australian scientists.

**Table 2:** Top 10 researchers based on votes and weighted votes

Rank	Researcher	Votes	Rank	Researcher	Weighted votes
1	Caroline Finch (F)	14	1	Caroline Finch (F)	128
2	*Male*	7	2	Jill Cook (F)	65
3	*Male*	7	3	Adrian Barnett (M)	57
4	Ian Frazer (M)	7	4	Ian Frazer (M)	55
5	Jill Cook (F)	7	5	Paul Glasziou (M)	53
6	Adrian Barnett (M)	6	6	*Male*	52
7	Julie Byles (F)	6	7	*Male*	51
8	Nicholas Graves (M)	6	8	*Female*	51
9	Paul Glasziou (M)	6	9	Rebecca Ivers (F)	42
10	*Male*	6	10	Julie Byles (F)	38

A few researchers did not respond to our request to use their name; hence, we only present their gender (in italics)

The number of votes by gender is in Table 3, together with the gender of recent ARC/NHMRC fellowship winners. There were many more votes for women than recent fellowship winners. The rate of votes for women was 1:32 times higher than the rate of female fellowship winners (95% confidence interval 1:20 to 1:45, *p* value <0.001). However, the difference may be due to Simpson’s paradox as fellowship schemes are stratified by field and some fields may have more female applicants [27]. If women are over-represented in the most competitive fields then a simple statistical comparison of the success rates for men and women will likely show a statistical difference even when no gender bias exists.

**Table 3:** Gender of vote winners and winners of ARC/NHMRC fellowships

	Votes		ARC/NHMRC	
Gender	*n*	Percent	*n*	Percent
Female	501	45	516	34
Male	617	55	1004	66
All	1118	100	1520	100

**Fig. 2:**
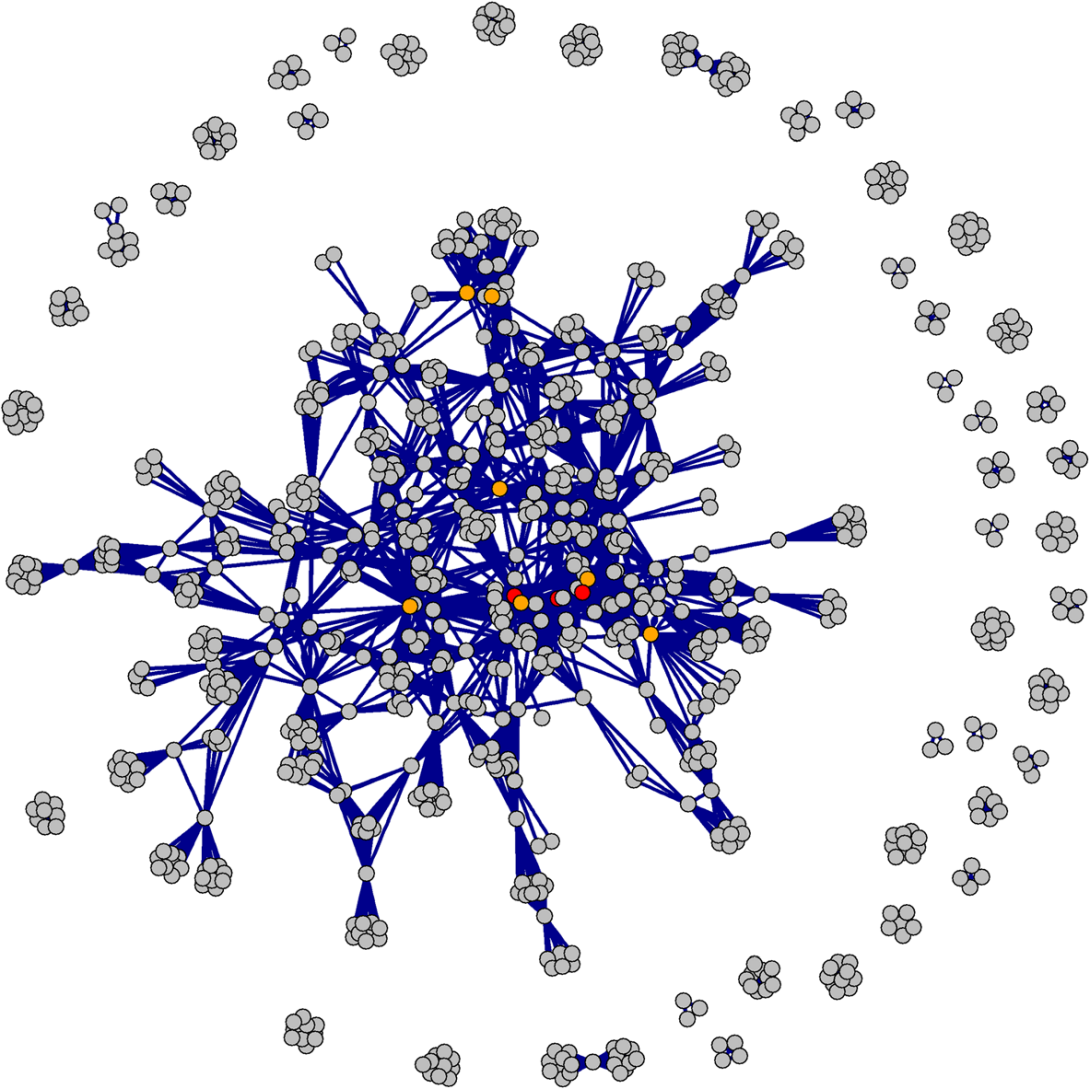
Network diagram joining researchers who were mentioned in the same ballot (*n* = 4004 connections). The top 10 researchers from Table 2 are in *orange*, and the study’s authors are in *red*

The network diagram of funding patterns in Fig. 2 shows that, not surprisingly, the top 10 researchers were in denser parts of the network. The study authors were also in a dense part of the network, which is not surprising given that we used our existing networks to distribute the survey. Also evident are the number of isolated ballots that have no connection with other ballots, as well as ballots on the edge of the dense network with just one or two connections. These isolated ballots were from researchers whose own networks were not well represented, and we would expect fewer “islands” with a larger number of ballots.

### Respondents’ comments

There were many negative comments about the idea of using democracy to award funding. Concerns were raised about vote rigging, lobbying and it becoming a popularity contest as typified by this quote:

I’m curious how this approach is intended to be anything other than a popularity contest, benefitting those who have already become ‘names’. Particularly, how will this help early career researchers, who are most disadvantaged by the current system?

Some also felt that it would favour bigger institutions and men, and would disadvantage mid- and early career researchers.

A number of respondents gave reasons why they did not vote. Many said they needed a list of names or that the candidate field was too large. Some said they did not know who was good or could not vote outside their field.

Positive comments were made about the potential time saving and the desire to try something new given the problems with the current system.

## Discussion

We tested the idea of a democratic system that would have almost no application costs and hence would save researcher time. Many researchers did not like the idea of using democracy and raised criticisms that we consider below.

### Lobbying

The potential problems of a democratic funding system would likely be the same as the political ones. Researchers may spend more time lobbying than on actual research, and the system may benefit scientists at larger institutions with more resources for lobbying.

As in politics, those researchers that are closest to the cut-off would have the greatest motivation to lobby, whilst highly successful researchers in a “safe seat” would not need to do much. We saw some evidence of well-known researchers doing well with some recognisable names in the top 10 (Table 2). However, such researchers often do well in the current funding system and very talented researchers are likely to be highly ranked by any system. Our previous research on project grants found that the top 9% of applications were always funded regardless of what peer reviewers were selected [7]. Quotes from peer reviewers have also talked about the ease of identifying high quality, for example from an interview with a panel member who had just completed a ranking exercise for the ARC: “In the field of 600 or so applications that the panel saw there were always 10% at the top of the field that stood out” [17]. If such researchers are always very likely to do well, and considering that such researchers are amongst the best in the country, then getting them to complete lengthy applications forms is a waste of valuable time.

Given the size of a national voting system, many researchers may decide that lobbying is futile. We estimated there would be almost one quarter of a million votes, which would likely create large gaps in vote numbers between the winners and losers. Bridging these gaps by lobbying would only be possible for those close to the funding line.

There are some potential benefits from researchers lobbying other researchers for votes. A great place to lobby would be at national conferences, and hence, these conferences would likely increase in prestige, providing a boost for national bodies and potentially increasing national collaborations. Secondly, the most effective lobbying would be done in the open in order to reach the greatest number of fellow researchers [23]. This would allow other researchers to openly disagree or question brazen claims and would also potentially increase the public’s engagement with research.

### Friendly voting

We found some evidence for a home institution benefit with an average extra 1.6 votes per ballot, and there were two ballots where researchers only voted for colleagues from their institution. This is not a large benefit and may reflect greater familiarity with local researchers.

We saw a potential friendly effect as two authors involved in the study appeared in the top 10. This is probably because the invitation to participate was via our networks and such a benefit should disappear in a national system. However, we note that both authors have current fellowships from the NHMRC, and so their appearance in the top 10 may also be a genuine recognition and not simply due to friendly voting.

Individuals working in big fields with many researchers are likely to get more votes than highly specialised fields where fewer people work. This may partly be a fair reflection of the research workforce and historical priorities. To avoid money simply flowing to the largest fields, votes could be stratified by field with high-level decisions made about the number of researchers to fund in each field. This would enable funding for smaller fields that were a national priority.

### Early career researchers

Many researchers were concerned that a voting system would be strongly biased against early career researchers who were not yet well known. A voting system relies on accumulated knowledge and therefore may not be suitable for awarding early career funding. However, early career researchers often struggle to compete in the current systems, again because of their lack of notoriety and reduced time to prove themselves. This lack of information will bedevil any funding system.

### Vote rigging

Researchers were concerned about vote rigging, with friends voting for friends or whole institutions voting for themselves. Such behaviour at the individual level would probably have no benefit given that we estimate a national system would have around a quarter of a million votes. At the institutional level, it would be hard to keep such behaviour secret given the number of people involved [28]. An online voting system would also know exactly who voted for who and could easily identify unusual voting patterns and impose penalties for institutions that try to fix the system. For example, the number of “friendly” votes for each institution could be examined (after adjusting for institution size and researcher quality based on the votes attracted by other institutions) and those institutions that were clear outliers could be investigated.

### Limited experiment

This was a limited experiment, akin to a phase I study of a new drug. Our aim was to get preliminary data on how a democratic system might work, and there are many questions about a democratic system that cannot be answered here but could be by a large trial. Ideally, a large trial would be a head-to-head study of two or more alternative systems (Table 1). Finding the better system would take significant time and effort as an ideal study would fund researchers using the competing systems and then prospectively compare their performance over five or more years.

The short voting times we observed may be an artefact of the experiment, and if real money were at stake then voters could take more time considering their votes and talking with colleagues, as well as spending time being exposed to lobbying. We would only need an increase of 38% per ballot (three additional minutes) for the voting system to take longer than the current peer review system. Concerns have been raised about national voting schemes to choose between competing charities, because of the time needed to lobby for votes and cast votes, much of which time is wasted when success rates are low [29].

### Related approaches

Voting has been suggested as a form of post peer review for journals [30] and as a way of democratising the peer review process [31]. It has also been used to award prizes in research, such as the “Peer Prize for Women in Science 2017” [32].

Two related studies have tried to use “the wisdom of the crowd” to allocate research funding [33, 34]. One approach is to give all researchers an equal amount of money but require them to pass on a fixed percentage of the previous year’s income to “other scientists whom they think would make best use of the money” [34]. Like a voting system, this would be an annual event that should be relatively quick to complete, and researchers who are well regarded by their peers would do well. The redistribution system would see researchers rewarded in proportion to their esteem, and the researchers held in the greatest esteem would also be trusted with more money to redistribute. A voting scheme could also give proportional rewards by allocating funding in proportion to votes.

Prediction markets were used to rank the esteem of UK research departments and did well with a small sample size of peer reviewers [33]. The principle is that a prediction market is better at getting reviewers to show their true beliefs. Similarly to voting, this is a quick process that relies on the reviewers’ knowledge built up over years of working in the field. However, the study ranked departments and may not be as useful for ranking individual researchers.

### Future research and policy

We would like to see further studies on alternative funding systems including a democratic system. Policies concerning peer review and funding are changing [35], and surprising ideas like removing all application deadlines [36] and awarding funding randomly are being used [22].

An ideal next step would be for a funding agency to award some funding using alternative systems (Table 1), including a democratic process, in order to get a more realistic picture of voting patterns and issues. If successful, the funding amount could be gradually increased over time so that the system could be improved under less testing conditions. Alternatively, a funding agency could test alternative systems alongside the traditional peer review and compare the outcomes. Alternative systems are unlikely to replace all research funding but are a potential solution for reducing the large amounts of time researchers currently spend on long applications from which they often get no return.

Changes to the system often provoke strong reactions from researchers, as there is a bias towards the status quo. We can imagine the reaction if the current system were democratic and we suggested that it change to an oligarchy of high profile researchers deciding who wins funding.

## Conclusions

In Australia, voting to award research fellowships would take less time than traditional peer review but would still require nearly 600 days of peer review spread over 37,000 reviewers. The total number of votes would be around one quarter of a million, which would make it very difficult for individual researchers to game the system. Many respondents raised legitimate concerns about a voting system that could not be answered by this preliminary study. The ideal test of alternative systems would be for a funding agency to trial a range of alternative systems using a small amount of real funding.

## Additional files

Additional file 1: Survey questions. (PDF 84 kb)

Additional file 2: Additional material. Example approach to participate; patterns of item missing data. (PDF 136 kb)

## Abbreviations

ARC: Australian Research Council
NHMRC: National Health and Medical Research Council
