# Supplementary material for: Using democracy to award research funding: an observational study
Source: Res Integr Peer Rev. 2017 Sep 15;2:16. doi: 10.1186/s41073-017-0040-0 (PMC5803583; doi:10.1186/s41073-017-0040-0)
Supplement: Supplementary file 1 — Survey questions. (PDF 84 kb) [file 41073_2017_40_MOESM1_ESM.pdf]

Democratic funding

Informed consent

PARTICIPANT INFORMATION FOR QUT RESEARCH PROJECTA democratic funding system QUT Ethics Approval Number 1500000073 RESEARCH TEAMPrincipal Researcher: Adrian Barnett, A/ ProfessorAssociate Researcher: Nicholas Graves, Professor Faculty of Health, Queensland University of Technology (QUT) DESCRIPTIONThis project is part of our group's research into the best way to allocate health and medical research funding.   The purpose of this project is to examine whether a democratic system of funding could be used to fund scientists. Your votes are a form of peer review that harness local knowledge for a low cost. By combining many votes it may be possible to identify good scientists nationally. Of course there are many ways that a funding system could go wrong. This initial study will examine the potentials and pitfalls of using democracy. For further details on our ideas read our article. You are invited to participate in this project because you are an Australian scientist.PARTICIPATIONParticipation will involve completing an anonymous survey that will take approximately 15 minutes. Questions include what Australian scientists you think most deserve funding and some basic information about you.   Your participation in this project is entirely voluntary. If you agree to participate you do not have to complete any questions you are uncomfortable answering. Your decision to participate or not participate will in no way impact upon your current or future relationship with QUT. If you do agree to participate you can withdraw from the project without comment or penalty. However as the survey is anonymous once it has been submitted it will not be possible to withdraw.EXPECTED BENEFITSThis project will not directly benefit you. If voting is shown to have sufficient agreement with the current system then there is the potential to save hundreds of years of scientific time in Australia.RISKSThere are no risks beyond normal day-to-day living associated with your participation in this project.PRIVACY & CONFIDENTIALITYAll comments and responses are anonymous and will be treated confidentially unless required by law.   Any data collected as part of this project will be stored securely as per QUT's Management of research data policy.CONSENT TO PARTICIPATESubmitting the completed online questionnaire is accepted as an indication of your consent to participate.QUESTIONS / FURTHER INFORMATION ABOUT THE PROJECTIf have any questions or require further information please contact one of the research team members below.Adrian BarnettNicholas Graves07 3138 601007 3138 6115a.barnett@qut.edu.aun.graves@qut.edu.auCONCERNS / COMPLAINTS REGARDING THE CONDUCT OF THE PROJECTQUT is committed to research integrity and the ethical conduct of research projects. However, if you do have any concerns or complaints about the ethical conduct of the project you may contact the QUT Research Ethics Unit on 3138 5123 or email ethicscontact@qut.edu.au. The QUT Research Ethics Unit is not connected with the research project and can facilitate a resolution to your concern in an impartial manner.Thank you for helping with this research project. Please keep this sheet for your information.By agreeing to participate in this study, you are agreeing that you: have read and understood the information provided in the Information to Participants section.have had any questions answered to your satisfaction.agree to participate in this online survey.understand that once you have submitted your responses, these cannot be withdrawn.

Please indicate your response to the statements above and consent to participate

- [ ] I agree and give my consent

We would like to know a little about you.

Which of the following best describes your current position?

- ○ Professor
- ○ Associate Professor
- ○ Researcher
- ○ Lab head
- ○ Technical staff
- ○ Lecturer
- ○ Postgraduate student
- ○ Administrative staff
- ○ Not working as a scientist or in research
- ○ Retired

Other

....

What is your broad area of research?

- ○ Basic science
- ○ Clinical science
- ○ Public health
- ○ Health services research
- ○ Mathematical sciences
- ○ Physical sciences
- ○ Chemical sciences
- ○ Earth sciences
- ○ Environmental sciences
- ○ Biological sciences
- ○ Agriculture and veterinary sciences
- ○ Information and computing sciences
- ○ Engineering
- ○ Technology
- ○ Built environment and design
- ○ Education
- ○ Economics
- ○ Psychology
- ○ Law
- ○ Creative arts

Other

....

Which funding agency do you apply to more?

- ○ NHMRC
- ○ ARC
- ○ NHMRC/ARC equally
- ○ Government
- ○ Other

Please name the 10 scientists currently working in Australia you think most deserve funding for future research. Naming fewer than 10 is fine. Please do not include prefixes like "Professor". Please write their first and second names, or their initials if you don't know their first name. Please also give their current institution. Example: "Albert Einstein" "QUT"

	Name	Institution (acronyms are fine)
1 (highest rank)	....	....
2	....	....
3	....	....
4	....	....
5	....	....
6	....	....
7	....	....
8	....	....
9	....	....
10 (lowest rank)	....	....

How long did it take you to think of the names?

....

What is your current institution?

- ○ Rather not say
- ○ Not working in Australia

Your institution (e.g., "University of Melbourne")

....

Do you have any comments on the idea of using a democratic funding system?

....

....

....

....
