# Supplementary material for: Using democracy to award research funding: an observational study
Source: Res Integr Peer Rev. 2017 Sep 15;2:16. doi: 10.1186/s41073-017-0040-0 (PMC5803583; doi:10.1186/s41073-017-0040-0)
Supplement: Supplementary file 2 — Additional material. Example approach to participate; patterns of item missing data. (PDF 136 kb) [file 41073_2017_40_MOESM2_ESM.pdf]

Appendix

Using democracy to award research funding: an observational study

Example e-mail approach

Subject: A democratic funding system

Dear fellow scientist

As a member of the Australian scientific community, we would like you to vote for the ten Australian scientists you feel most deserve funding. To vote you complete an anonymous survey that should take around **15 minutes of your time**.

We want to see if it's feasible to replace some of the current funding schemes using a voting system. The great advantage of a democratic funding system is that it is very low cost, compared with the lengthy applications that we must currently submit. In this preliminary research we are interested in the feasibility of using votes as a form of peer review.

You are invited to participate in this research because you are a scientist working in Australia.

If you want to read more about our ideas then read the Conversation article here.

To participate in our survey, please click this link: **http://survey.qut.edu.au/f/182927/2015/**.

This study has been approved by the QUT Human Research Ethics Committee (approval number 1500000073).

Thanks for your time.

Regards

Adrian Barnett PhD | Senior Research Fellow, PhD

Institute of Health and Biomedical Innovation (IHBI) | School of Public Health & Social Work
Queensland University of Technology (QUT) | 60 Musk Ave, Kelvin Grove, QLD 4059 | Australia
T: 07 3138 6010 | F: 07 3138 6030 | E: a.barnett@qut.edu.au | www.ihbi.qut.edu.au | @aidybarnett

Example tweet

Could we use democracy to fund researchers? We want your votes http://survey.qut.edu.au/f/182927/2015/

Patterns of item missing data

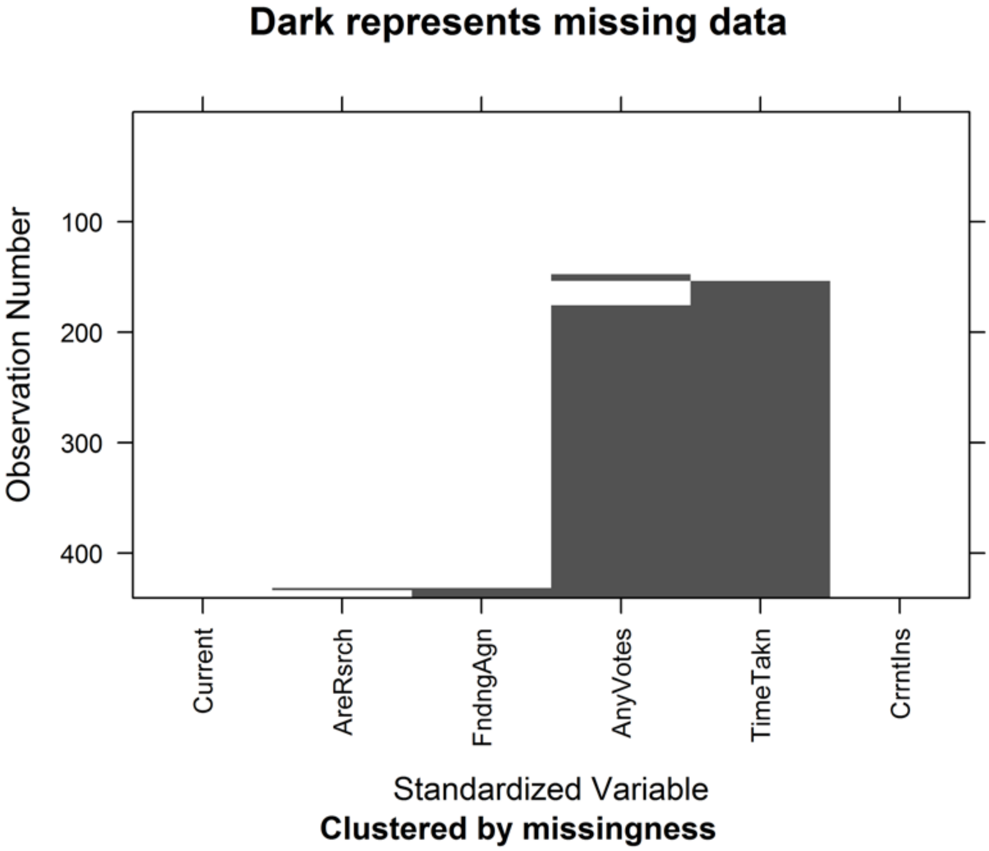

Table of item missing counts from 440 responses

Variable label	Variable	Number missing (%)
Current	Which of the following best describes your current position?	0 (0)
AreRsrch	What is your broad area of research?	2 (0.5)
FndngAgn	Which funding agency do you apply to more?	9 (2.0)
AnyVotes	Any eligible votes?	271 (61.6)
TimeTakn	How long did it take you to think of the names?	287 (65.2)
CrrntIns	What is your current institution?	0 (0)

Table of respondent characteristics by whether they voted or not

		Voted			
		No		Yes	
Variable	Category	N	%	N	%
Institution	Professor	36	13.3	34	20.1
	Associate Professor	24	8.9	18	10.7
	Researcher	98	36.2	60	35.5
	Lab head	4	1.5	7	4.1
	Technical staff	7	2.6	1	0.6
	Lecturer	37	13.7	14	8.3
	Postgraduate student	51	18.8	24	14.2
	Other	14	5.2	11	6.5
Area of Research	Basic science	18	6.6	11	6.5
	Clinical science	22	8.1	9	5.3
	Public health	75	27.7	47	27.8
	Health services research	35	12.9	24	14.2
	Mathematical sciences	5	1.8	1	0.6
	Physical sciences	6	2.2	3	1.8
	Chemical sciences	3	1.1	5	3.0
	Earth sciences	2	0.7	3	1.8
	Environmental sciences	13	4.8	7	4.1
	Biological sciences	38	14.0	26	15.4
	Agriculture and veterinary sciences	2	0.7	1	0.6
	Information and computing sciences	2	0.7	1	0.6
	Engineering	7	2.6	1	0.6
	Technology	0	0.0	1	0.6
	Education	3	1.1	1	0.6
	Economics	2	0.7	3	1.8
	Psychology	22	8.1	11	6.5
	Law	0	0.0	1	0.6
	Creative arts	0	0.0	0	0.0
	Other	14	5.2	13	7.7
Funding agency	NHMRC	110	40.6	82	48.5
	ARC	51	18.8	30	17.8
	NHMRC/ARC equally	36	13.3	23	13.6
	Government	31	11.4	15	8.9
	Other	34	12.5	19	11.2
	All	271	100.0	169	100.0
